# Supplementary material for: Fast and Sensitive Detection of Soil-Borne Cereal Mosaic Virus in Leaf Crude Extract of Durum Wheat
Source: Viruses. 2022 Dec 31;15(1):140. doi: 10.3390/v15010140 (PMC9866084; doi:10.3390/v15010140)

Figure S1.

LAMP temperature gradient tests from 60 to 65°C, performed on cDNAs from SBCMV-infected wheat plants. Experiment performed in the CFX instrument. Green = SBCMV positive control; violet = negative control; blue = no template (water); RFU= Relative fluorescence units

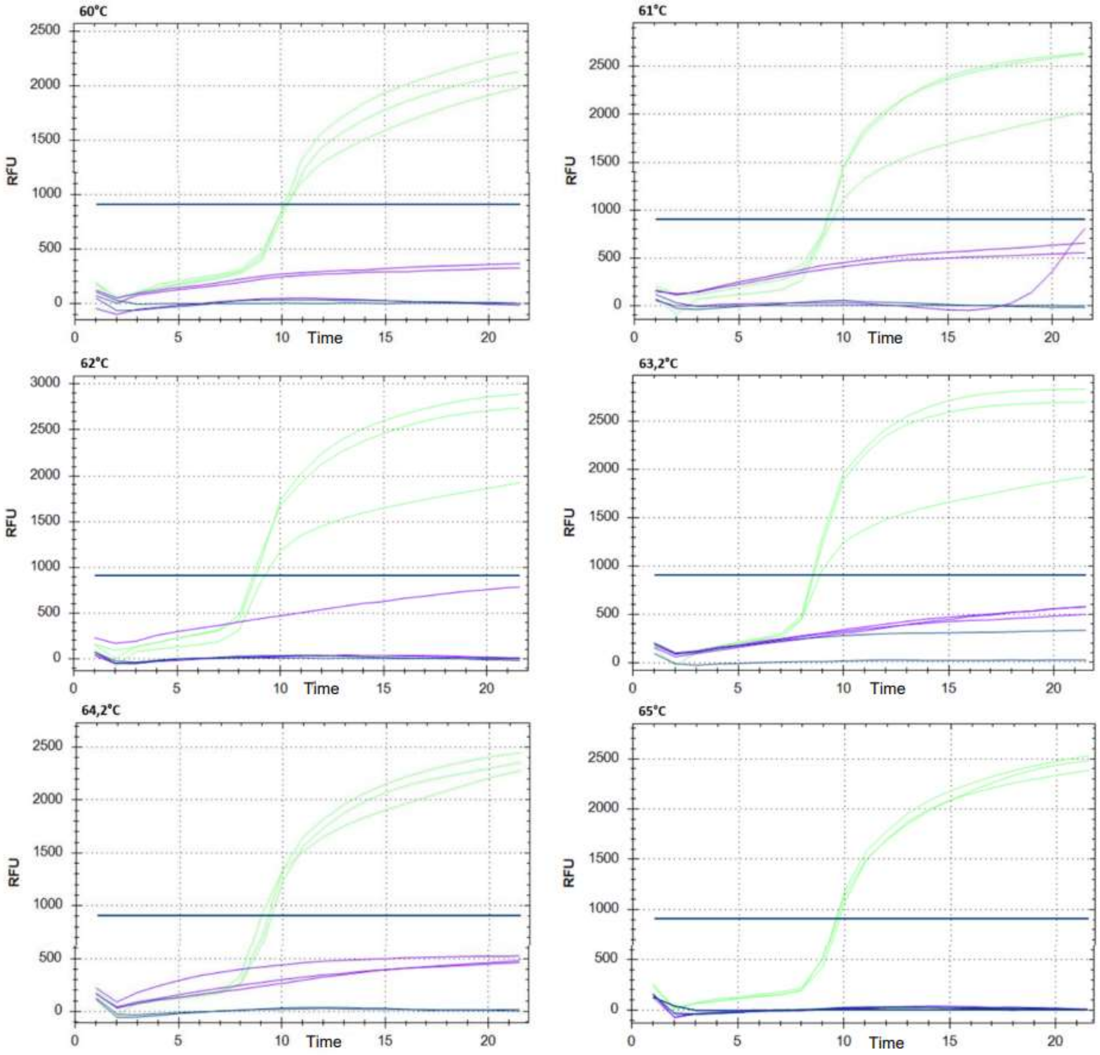

Supplement: Supplementary file 1 [file viruses-15-00140-s001.zip › viruses-2091114-supplementary.pdf]
